# Hospital and Institutional News

**Published:** 1916-11-11

**Authors:** 


### HOSPITAL AND INSTITUTIONAL NEWS.

### THE KING AND QUEEN AT SUSSEX HOSPITALS.

Their Majesties the King and Queen visited two large convalescent camps at Shoreham and Eastbourne respectively on November 3. Although the visits were private, and instructions were given that flags and bunting should not be displayed, their Majesties received a most hearty and loyal welcome from the inhabitants of both these Sussex towns; the route to Shoreham Camp was lined by Canadians. At the camp the massage and electrical department received careful attention from the Royal visitors, as did also the "Welcome" hut. Exhibitions of physical drill, bomb-throwing, bayonet-fighting, and trench-fighting were included in the programme. Later in the day the similar camp at Eastbourne was visited, and here, too, an exhaustive tour of the camp was undertaken. The "Almeric Paget" Electrical and Massage Institute, the theatre, billiard-room, cookhouse, kitchen, and quartermaster's stores were amongst the departments which their Majesties inspected. The keen interest which both the King and Queen always take in the welfare of all their subjects, and especially of those who are or have been invalided from the fields of war, was shown by the frequent questions that were asked. At the conclusion of the tour their Majesties expressed themselves as highly delighted with all they had seen.

### HONOURS FOR GARDELEGEN HEROES.

The precedent of Wittenberg has been exactly followed in the matter of decorations for the heroic doctors of Gardelegen Camp, where German infamy, brutality, and cowardice sounded depths as deep as those of the former place. That is to say, the senior officer of the Royal Army Medical Corps who worked among the typhus patients has been awarded the C.M.G.; while the two junior ones receive a D.S.O. apiece. Their names are Major P. C. T. Davy, and Captains A. Scott-Williams and A. J. Brown respectively, and the hospital world, as well as the medical profession and the whole nation, holds them as high in honour as it does Major Priestley and his comrades of Wittenberg. Just as at this latter camp, there was splendid heroism displayed by men of the rank and file, duly acknowledged by the bestowal of appropriate decorations, so too, it is to be expected that those who co-operated with Major Davy as orderlies and helpers at Gardelegen will in due course obtain recognition similar to that of their comrades of Wittenberg.

### CARDIFF'S GREAT DAY.

The visit of Mr. Walter Long, President of the Local Government Board, to Cardiff on November 3 to lay the foundation-stones of the new chapel and maternity wards, which are the latest of Mrs. John Nixon's great gifts to the King Edward VII.'s Hospital, marked a notable occasion. It was the hospital's happy day, for the wells of generosity were overflowing. Colonel E. M. Bruce Vaughan, whose name is a veritable organiser of victory in the field of charitable enterprise, was able to announce another princely benefaction—namely, a gift of £50,000 from Commander Sir Edward Nicholl, R.N.R., for the erection of a nursing home and maternity hospital. This gift, which is only second in importance to Sir William James Thomas's magnificent donation of £100,000 for building the laboratories of the Welsh National School of Medicine, has attached to it two conditions, both of a businesslike character. One is that the site of the home and hospital shall be freehold, given by the ground landlord or purchased from him at a cost of £1,500; and the other is that the income needed, estimated at about £6,000, shall be assured and 75 per cent. of it secured by the time the building is complete, the balance of £1,500 to be found by annual subscriptions or endowment.

### SIR EDWARD NICHOLL'S AMBITION.

Sir Edward Nicholl, who quite recently gave £10,000 in order to furnish thirty-two surgical beds at the hospital, told the assembly at Cardiff: "I have had an ambition all my life to have a lasting memorial when I am boxed up. If some poor girl—I care not whether she be Catholic or Protestant, Hindoo or Mahomedan—comes to the hospital she is not to be turned away from the front door. If some poor girl is in trouble and wants assistance—never mind who she is, or what she is, or where she comes from—she must have the best attention. If this and the other few conditions are complied with, then the building can be completed in three or four years, and you may count on me to foot the bill." In the course of the proceedings donations were announced of 1,000 guineas from Sir W. J. Tatem; 1,000 guineas from Mr. J. H. Mullins (Llanishen), in memory of Captain R. H. Mullins, killed in action in July last; £270 from Sir W. J. Thomas for the organ in the new chapel; £100 from Major and Mrs. Cornelius Griffiths for the pulpit; £250 from Mr. F. R. Crutchley, and £100 from Miss Talbot, besides a number of others of smaller amount.

### A MINISTRY OF PUBLIC HEALTH.

Mr. Walter Long, President of the Local Government Board, delivered on the same occasion an address which contained a significant reference to a Ministry of Health. Mr. Long regrets very much that our laws are not such as to concentrate the whole of the medical supervision of old and young alike in the hands of one great Government Department, which alone should be responsible. He hopes, however, to see the time when there will be a great central single Public Health Authority. This portion of Mr. Long's speech stopped short at the most interesting point, and we regret that the President of the Local Government Board did not deal in a rather more exhaustive way with this important question of centralised health administration.

### A HOSPITAL ROMANCE.

Before quitting the subject of Cardiff, the King Edward VII.'s Hospital, and its splendid list of generous friends, we cannot refrain from mentioning a small item of news which appeared in the press on November 7. Personalities of a private nature are seldom to be found in these columns, but there are special reasons in the present case why congratulations should be offered to Sir William James Thomas on the announcement of his engagement to Miss Mary Maud Cooper, the assistant matron of the King Edward VII.'s Hospital. Readers of The Hospital are well aware of the many munificent donations of this Welsh coal-owner and philanthropist to voluntary hospitals. The crowning gift of £100,000 for the erection of buildings for the Welsh Medical School was only one of a long series of splendid charities, not all confined to Cardiff, totalling, it is said, a quarter of a million pounds. That Sir William and his future wife will continue to display the same interest in the King Edward VII.'s Hospital, and that they may live long and happily, will be the wish not only of the Cardiff institutional world, but of all who know either of them and their records.

### THE DEVELOPMENT OF THE WEST LONDON HOSPITAL.

Under the title "In Earlier Days" Mr. Percy Dunn has for two years past been contributing to the quarterly *West London Medical Journal* his reminiscences of his professional connection with the West London Hospital as ophthalmic surgeon. Hitherto these reminiscences have been largely personal and anecdotal; but in the concluding instalment, just published, he branches off into topics more especially affecting the management of the hospital. Mr. Dunn deals with the somewhat delicate subject of the tendency of the board at this hospital to override the recommendations of the medical committee in regard to staff elections. While justly pointing out that the board alone has the legal right to make such appointments, he admits in another passage that in practice it has been the chairman who has imposed his will on the other members of that body, and has thus on his sole initiative controlled staff elections. This statement confirms what was long ago common gossip about this point. Although no hospital board should submit to dictation from its medical committee about new staff appointments, it is equally undesirable that it should allow the personal predilections of its head to sway its decisions. Perhaps matters are different now at the West London from what they were "in earlier days."

### THE NEEDS OF THE OPHTHALMIC DEPARTMENT

Mr. Dunn passes on to an eloquent plea for the thorough reorganisation of the ophthalmic department at the West London Hospital. His account of its shortcomings and lack of facilities certainly enables him to make a strong case; the marvel is that so much good work is and has been done there in so disadvantageous an environment. The ophthalmic department of the hospital is, he declares, capable of unlimited expansion, if the deficiencies of its organisation and equipment are remedied. The ophthalmic out-patient department is badly planned and inadequate in many different ways. The in-patient department consists of four beds. There is no help provided for the visiting ophthalmologists, who have to drudge wearily at refractions in depressing surroundings. To the anticipated objection that no funds are available for the construction of the male and female ophthalmic wards and for the reorganisation of the O.P. department for which Mr. Dunn pleads, his answer is that a special appeal for this purpose ought to be taken in hand. Whether the authorities of the hospital have any more complete answer to this friendly critic remains to be seen: *prima facie* he seems to have made out a very strong case.

### AN IMPROVEMENT SCHEME AT ST. GEORGE'S.

At their next General Court the Governors of St. George's Hospital will have submitted to them for consideration plans for a radical reconstruction of the out-patient and other departments, which have been approved by the house committee. There is, we gather, no present intention to take active steps to carry out this scheme until after the war; but it has been thought advisable to use the opportunity for carefully maturing the plans and getting all necessary approvals. The reasons put forward by the house committee for their dissatisfaction with the present condition of the hospital are stated with force and brevity in the report. The buildings have, admittedly, been for years inadequate for the work that is carried on in them. The committee mention especially the cramped out-patient and casualty departments, which have been condemned by representatives of King Edward's Fund and are quite indefensible. Great inconvenience in administration is also caused by the housing of the nurses at a distance from the hospital. The various proposals of the last ten or twelve years for removing the hospital to another site have naturally deterred the committee from advocating heavy expenditure to bring these departments up to date; but now that it is finally recognised that removal is out of the question avoidable delay can no longer be brooked, and hence the formulation of the present scheme.

### THE DETAILS OF THE PROPOSALS.

On some future occasion we shall hope to examine critically the new plans. All that can be said for the moment is that they appear to provide accommodation for some of the hospital's most pressing needs; to facilitate and centralise administrative control in a most praiseworthy way; to place the out-patient department (which is certainly the most out-of-date and imperfect part of the existing buildings) on a satisfactory footing; and to improve very greatly the accommodation for the resident medical officers, as well as to provide for the housing of the nursing staff on the premises instead of at a distance of a mile. All this is to be accomplished by the erection of a new building on the site of the four houses in Knightsbridge between the "Cottage"

and the Tube station. This building is to be so arranged that it may form part of any new hospital to be built on the present site. It will consist of basement, ground floor, and six floors above, and will extend from the west end of the present Wellington Ward to the Tube station, with a wing at right angles extending to the medical school. By a rearrangement of the rooms on the ground floor and basement of the present buildings, rendered possible by the proposed extension, it will be possible to provide a midwifery ward of twenty-two beds. All the resident officers will be housed on the first and second floors of the new buildings; and the top four floors will be allotted to the nurses, who will have a separate entrance in Knightsbridge, and will be provided with a lift. The electrical department will be moved from its present quarters to the basement; and the rooms now occupied by it will, with the present secretarial offices, be made into a ward to replace Wellington Ward, which will form part of the new casualty department. The administrative offices will be along the back of the ground floor of the hospital, on each side of the board-room, where the casualty receiving rooms are at present.

### A CURRENT FALLACY ABOUT THE ROEHAMPTON HOSPITAL.

Mr. C. H. Kenderdine, Honorary Secretary and Treasurer of Queen Mary's Convalescent Auxiliary Hospitals, Roehampton, has found it necessary to issue a public statement to correct the notion, which prevails somewhat generally, that the artificial limbs which are provided for sailors and soldiers at Roehampton cost about £50, towards which the men themselves have to pay £40. He says that whatever limbs the surgeons order are fitted irrespective of the cost, and that the only men who have to contribute anything are those who, contrary to the surgeons' advice, insist upon having arms or legs which the surgeons consider will prove of no practical value to them. It is not perhaps widely known that all repairs, readjustments, and renewals of artificial limbs for sailors and soldiers are paid for by the State.

### KING MANOEL'S CAMPAIGN.

Accompanied by Colonel Jones, Director of Military Orthopædics, King Manoel has been making a tour of the Scotch military hospitals in his capacity as representative of the Red Cross Society in relation to orthopædics and the wounded. His aim has been to help the completion of fully-equipped departments at the different hospitals, and further to extend their existing work by inaugurating a series of workshops for the training of the patients on the lines of which much has been heard in London and elsewhere. King Manoel enforced his appeal for the institution of workshops by stating that if the Red Cross would supply and equip them in the different hospitals the War Office would guarantee their maintenance. For he was convinced that the agreement which had been made as regards England would be equally applicable to Scotland once the workshops had been set up.

### THE MANCHESTER AND SALFORD LOCK HOSPITAL.

Presiding last week at the biennial meeting of the Manchester and Salford Lock Hospital, Mr. Wilfred Becker touched upon the position of such institutions under the Local Government Board proposals for the treatment of venereal diseases. The fact that there are only four Lock hospitals in the country—at London, Manchester, Glasgow and Dublin—probably accounts for the fact that no reference is made to their co-operation in the circular issued to local authorities by the Local Government Board. Mr. Becker said that though the Royal Commission and the Local Government Board were in favour of treatment at general and not special hospitals, it would be unfortunate if the four existing Lock hospitals, which were particularly adapted for the work and had specially experienced staffs of medical officers, were placed in the melting-pot. Such a fate did not impress Major Wilson, one of the honorary medical officers, as a likely one, for he urged an immediate extension of the hospital so as to be prepared for the large increase in the number of patients at the end of the war. The removal of the hospital to a more modern and adequate building on a site nearer Manchester University, together with a change of name, was suggested.

### AN ADDITION TO HALIFAX WAR HOSPITAL.

The request of Lieutenant-Colonel Woodyatt, administrator of Halifax War Hospital, for the provision by public subscription of an installation of Zander apparatus for the treatment of stiff joints and muscles, has been generously answered. The new equipment, which cost some £300, has now been added to the institution, and, placed under the direction of three trained masseuses, is installed in what was formerly the lecture-room. This mechanical treatment forms part of the majority of war hospitals where orthopædic patients are received, and should prove equally valuable at the Halifax institution. The invention of a Swedish doctor, whose name it bears, it has been largely employed since the war gave to orthopædics a new importance, at least in England.

### SHOULD A HOSPITAL CHAPLAIN DO ALMONER'S WORK?

A somewhat ambiguously worded advertisement, issued by order of the Weekly Board of the Middlesex Hospital, appears to foreshadow the possibility of a new development in hospital administration. The board are inviting applications for the post of chaplain to the hospital. Candidates must be ordained priests of the Church of England, unmarried, and not more than forty-five years of age. It is evidently intended that the appointment shall be a whole-time one, for board and residence in the hospital are provided, as well as a salary of £200 a year. Further, it is stated—and this is where the ambiguity comes in—that " an additional allowance will be made if the work of almoner is attached to the office." If there is any intention on the part of the Middlesex Hospital Board to conjoin the two posts of chaplain and almoner, the result will certainly be watched with interest in institutional circles. That some such scheme is under consideration is suggested by the condition that candidates must be under forty-five. The purely religious duties of a hospital chaplaincy can quite well be carried out by a man beyond this age; but if the duties of almoner are to be superadded, the age limit is comprehensible and necessary.

### A LINCOLNSHIRE TUBERCULOSIS SCHEME.

A CAMPAIGN against tuberculosis was being inaugurated by the Lindsey (Lincolnshire) County Council when its further progress was suddenly cut short by the outbreak of war. However, the dispensary scheme was continued with a depleted staff, in order that the organisation might be kept in being. Dr. R. A. Glegg, the county medical officer, in his annual report reviews what has been done to cope with the disease, and remarks that the open-air shelters were found to be of the greatest value in treatment, and also as a means, in many cases, of removing from overcrowded houses patients who would otherwise have been much more liable to convey infection to others. Dr. Glegg emphasises the necessity of impressing upon the authorities and magistrates the urgent need of pure, unadulterated milk, clean and free from the germs of tuberculosis.

### PUBLIC FUNDS AND DOUBTFUL METHODS.

FROM time to time it is our duty to remind the organisers of charitable funds that the desire to raise money, even for the best purposes, is apt to run riot owing to the thoughtlessness of people who fancy that the cause of charity justifies every method. At the present moment it is reported that a large money prize is being offered in a guessing competition, the proceeds of which, accruing from the purchase of the shilling entry forms, are to be devoted, it is understood, to Red Cross funds. This is an instance of the way in which zeal may outrun discretion, even in the best cause, for the proposed competition is degrading to the cause of Red Cross work, and may be condemned not only on this ground, but in view of the fact that the prize, which is stated to be £5,000, will render the "administrative expenses" too high, whatever be the total realised by it. It is a matter for regret that the Red Cross authorities should lend any countenance to this ill-considered, if well-intentioned, scheme, and we hope that it is not too late for them to repudiate all connection with it. Such methods do more to disgust intelligent persons with voluntary giving than anything else could do. If people are to subscribe to the Red Cross only on the chance of putting money into their own pockets the cause of charity is already dead, and such methods as this can only first degrade it.

### A NEW MEANING TO "AFTER-CARE."

THE futility of a sanatorium "benefit" which gives a consumptive three months' institutional treatment and then throws him back to his old condition (if, indeed, he is still able to get employment at all), has become too glaring to continue without protests and attempts at reform. The protests we have had in profusion. The attempt at reform is beginning to be made. Various borough and county insurance committees—those of Bristol, the West Riding, and Worcester are three—have adopted resolutions urging the provision of "after-care treatment in farm colonies for tuberculous insured persons who have received treatment in an institution." Here, at any rate, is a concrete proposal; and if opinion in favour of it spreads there is some chance that a new meaning may be given to after-care, and that it too may grow to something more than domiciliary visiting. Institutional after-care in farm colonies is worth an experiment, and we hope that every insurance committee will consider the possibility of joining in a demand for its inception. Without joint action by the separate committees the scheme has no chance of official or central support.

### THE GERMANS AND NURSE CAVELL.

THE exhibition at the Mansion House of the proclamations issued by the German military authorities to the inhabitants of French and Belgian towns now under their occupation includes one of special interest to hospitals. This is the proclamation which was posted in Brussels over the signature of General von Bissing—a warning-notice in which the name of Nurse Edith Cavell occurs with five others in a list of persons "condemned to death for organised treason." The proclamation states that in two cases, of which Nurse Cavell's was one, sentence had already been executed.

### A NATIONAL INSTITUTE OF MOTHERCRAFT.

THE National Association for the Prevention of Infantile Mortality proposes to extend its already wide sphere of influence. A sub-committee has been formed to deal with the proposal to found a National Institute of Mothercraft, to comprise an ante-natal clinic, an infants consultation centre and clinic, a school for mothers, a day nursery, a nursery school, and an observation ward for ailing babies. It will also include a reference and lending library, lecture halls, a museum, and a permanent and travelling exhibition. Ample opportunity will be afforded for the study and investigation of all problems relating to maternity and infancy, including laboratory research in the dieting of infants generally. The provisional committee includes the Right Hon. A. H. D. Acland, Mr. Benjamin Broadbent of Huddersfield, Dr. D. Forsyth, the Medical Officers of Health for Glasgow, Liverpool, Huddersfield, and Birmingham, Dr. Eric Pritchard, Dr. Amand Routh, and Dr. Mary Scharlieb. The Duchess of Marlborough, who is acting as treasurer, has opened an office at Sunderland House, Mayfair, where the preliminary work of organisation will be carried out.

### THE EDITH CAVELL HOMES OF REST FOR NURSES.

THE Council of the Edith Cavell Homes of Rest for Nurses, whose scheme was mentioned in these pages a month ago (THE HOSPITAL, October 14, 1916; p. 21), is pushing forward with its proposals and its appeals for support to carry them out with commendable promptitude and energy. Lady Haig has issued a letter which contains a most admirable suggestion, and one to which wide publicity should be given. She thinks, and beyond all question thinks rightly, that many officers and men of the Army who have benefited by the unselfish ministrations of their nurses are anxious to give practical expression to their gratitude according to their ability; and she suggests that they could not do so in a more ideal way than by subscribing to this scheme for providing Homes of Rest for nurses worn out by overwork or prostrated by illness. It is further stated, in a circular which accompanies Lady Haig's letter, that the house near Haslemere, which has been already offered as a gift to the Council on condition that a sufficient endowment fund can be raised, is at Coombe Head, near Invall Common and Hindhead. It is 700 feet above the sea, and stands in three and a half acres of grounds. To convert it to the purposes of a Home for Nurses and to endow it a sum of £30,000 is required.

### CUTTING THE RED-TAPE KNOT.

So much did a gentleman, who had been collecting eggs for hospitals and selling the flowers in his garden for the benefit of the Red Cross, object to being registered under the War Charities Act that he is reported to have spent his time in trying to evade registration. He is compelled to give up charitable effort on his own behalf, so long as he refuses to register; but, making use of his position as a church officer, he has considered the possibility of placing a Red Cross offertory-box at the church door. Any funds raised by this means would be under the control of the church officers until paid over to the Red Cross authorities. Such a device, however, puts an end to the objector's individual efforts as thoroughly as it does to the need for registration. It is therefore but an evasion of the issue, which has its interest as an instance of our hereditary hatred of officialdom.

### WAR CHARITIES' ACCOUNTS.

It will be remembered that attention was called in these columns to the rather unreasonable requirements of the Charity Commissioners in respect of the rendering of war charity accounts every three months, and to the difficulty of finding auditors and accountants to carry out such work. We learn that a concession has been made to meet the general dissatisfaction caused by this regulation, and that now "duly audited accounts of every registered charity shall be sent to the registration authority at least once in every period of twelve months, but the registration authority, with the consent of the Commissioners, or the Commissioners, may call for such accounts at any time." Evidently any unnecessary calling for accounts will not be encouraged, and a war charity which is asked for a statement in between times will gather that there is something in the wind. A second amendment of the original regulation deals with gifts in kind; these must be carefully recorded but need not be valued for accountancy purposes. An official list of war charities registered up to October 21 can now be obtained through the booksellers.

### SOME NOVEL COLLECTING SCHEMES.

It is difficult to conceive, in view of the varied and multitudinous efforts to raise funds for various charities at the present time, of anything new in the way of a street collection or Flag Day. Two efforts, however, of a somewhat novel character have recently been brought to our notice: the one organised by the Printers' Medical Aid and Sanatoria Association, and consisting of a "Match Box" Day; the other, an effort by the residents of Wimbledon to raise funds for an ambulance motor-boat by a house-to-house collection of gold and silver trinkets. It is stated that the latter has met with such good success that an amount of something like £1,200 has been raised.

### THE REMUNERATION OF DOCTORS AT VENEREAL CLINICS.

The Council of the British Medical Association has issued a letter to the secretaries of divisions and branches laying down a tariff of fees and salaries for doctors appointed as whole- or part-time medical officers at clinics for the treatment of venereal diseases. This tariff is, of course, merely a suggestion, not a mandate; but it is to be submitted to the next representative meeting, and may thereafter be supported by the not inconsiderable power of the Association. There is no need to discuss here in detail the table of fees and salaries suggested as the minimum. They are certainly generous, and may be criticised as over-generous by those who habitually try to screw down the remuneration of any medical servant of the State. There are, however, two considerations which are relevant to counterbalance such a criticism. One is that doctors who devote themselves to the task of stamping out these loathsome plagues deserve to be well paid for it; the other, that unless able men are secured, the clinics will fail to do what is expected of them. In its turn the medical profession must see to it that really competent men are supplied, not the type that often rushes to take up any new kind of work through having failed to get on at any of the old kinds. Given good men and good pay, we expect to see a great future for the anti-venereal crusade.

### PROVISIONS FOR THE HOSPITALS.

The difficulties are increasing for many of the governing bodies of hospitals to obtain adequate supplies of provisions. This has been more particularly noticeable so far as sugar is concerned, and it behoves the Government to see that these institutions, more especially the military hospitals, have their supplies. At the Southwark Military Hospital the local Board of Guardians were unable to secure a regular supply, and Major H. W. Bruce, who is in charge as Medical Superintendent, communicated with the Royal Commission on the Sugar Supply, with the result that the hospital was allotted a weekly supply of eight hundredweights. One firm was selected to send the goods, the price being fixed by the Royal Commission. As to the quality, those in authority can say nothing. They have simply to take whatever is sent, without question. It remains to be seen how this arrangement will work, but at all events the hospital is sure now of getting a regular supply of this commodity. If similar Government action was taken with regard to other necessities, some of the difficulties of having a regular stock and supply of provisions would be overcome.

### THE "REP. MIST." FORMULA.

The London Insurance Committee has issued a letter to all chemists on the panel regarding the filling of prescriptions when only the formula "Rep. Mist." is used. The Insurance Committee were in favour of the total prohibition of the use of this term in prescribing, whilst the Panel Committee desired its use continued under certain conditions. It appears that the respective committees could not agree on an amicable solution of the problem, and it was therefore referred to the Insurance Commissioners for settlement. The Insurance Commissioners think that the use of the term is open to objection and ought to be discontinued, but they have decided that for the war period and until three months after peace is declared "repeat" prescriptions may be issued on certain clearly defined conditions. The Commissioners, in giving the decision, state that they reserve the right to alter it at the end of the present year, if they are satisfied by evidence that the rules set forth have been disregarded by practitioners on the panel to any great extent. Unless the insured person specifically requests a copy (in full) of the prescription requiring renewal, the free choice of obtaining his medicine from *any* chemist on the panel is debarred.

### A CURIOUS ARGUMENT AGAINST CLOSING AN IRISH WORKHOUSE.

The Irvinestown Guardians have decided to close their workhouse and fever hospital. The proposal was supported by figures which showed that last year the average number of inmates under care was twenty-nine; and that the cost of maintaining them, apart from officials' salaries, worked out at £1 a week each. It was also shown that those for whom the Guardians are responsible can be boarded out for seven shillings a week each, and that altogether a saving of well over £1,000 a year can be accomplished by the proposed change. A very curious argument was brought forward against the new scheme, though it did not avail to cause its rejection. This argument was that "the poor paupers would better be put against a wall and shot than sent away to a strange place and perhaps be badly treated." It may be hoped that Irish Poor-Law administration is not at quite so low an ebb as the speaker appears to believe. Perhaps, and more probably, his vehemently worded plea was merely a piece of Irish exaggeration, not meant to be taken seriously.

### A SCOTTISH HOSPITAL GAZETTE.

The November issue of the *Craigleith Hospital Chronicle* is mainly interesting for its promise of an elaborate Christmas Number, a promise which interests those who remember the two previous numbers associated with Christmas Day. In addition to the numerous articles which the editor manages to extract from past and present members of the staff and patients, there will be, we learn, several contributions from well-known writers, and the *Chronicle* is wise in its insistence first on matter worth reading, and secondly on securing as much of this as possible from those associated with the institution itself. Articles of general interest in the current issue include one on "Abbotsford," the home of Scott, by "S. B M.," and a further instalment of "A Nurse's Notes from Serbia," by "M. T. F.," in which the writer describes a voyage on the Red Cross ship *Erin*, once Sir T. Lipton's private yacht. "Not very long after our interesting and delightful voyage," concludes the author, "the *Erin* was unreservedly handed over by Sir Thomas to the Admiralty, and returned to the Mediterranean as the armed yacht *Ægusa*, and in May was sunk, just after she had rescued the crew of the *Nasturtium*." If a Gazette is any index of the attractiveness of the hospital from which it comes, and so diverse are these publications that the character of each hospital may well be reflected in its periodical, the 2nd Scottish General Hospital should be a delightful place.

### THIS WEEK'S DRUG MARKET.

A considerable number of price changes, for the most part of no great importance, have taken place; but on the whole the drug market continues quiet. An advance in the price of morphine was not unexpected in view of the rise in the value of opium, but up to the present makers have not changed their quotations for the alkaloid. Atropine has been reduced in price, the reason being that another important London firm of chemical manufacturers, having obtained supplies of the raw material, is producing the alkaloid; the reduction amounts to something over 10 per cent., but it is hoped that this is only the beginnning of a steady decline in the price of a drug the high cost of which has been severely felt, especially by ophthalmic hospitals. More business has been done in quinine during the past few days, and the price of Continental brands, which had been gradually sagging, is now steadier. Epsom salts is rather dearer. In synthetic drugs there are few changes; the demand for acetylsalicylic acid is not quite so strong, but the price is maintained; barbitone and phenacetin are still scarce; acetanilide is dearer; salol is lower. Tartarated antimony is cheaper. Lycopodium is in more plentiful supply at lower prices. The downward tendency in the prices of tartaric acid and citric acid continues.

### TO OUR READERS.

**Contributions are specially invited from any of our readers to these columns. They should deal with topical subjects and news. They must be authenticated for the information of the Editor only. The minimum payment if published is 5s. There is no hard-and-fast rule as to space, but notes of about twenty lines in length are preferred.**

